# Nanomechanics of phospholipid LB film studied layer by layer with AFM

**DOI:** 10.1186/s13065-014-0071-2

**Published:** 2014-12-10

**Authors:** Yinli Li, Changjiang Zhu, Jichun Zhu, Hao Liang, Dong Chen, Huiling Zhao, Bo Liu

**Affiliations:** Institute of Photo-biophysics, School of Physics and Electronic, Henan University, Kaifeng, 475004 Henan PR China; College of Software, Henan University, Kaifeng, 475004 Henan PR China

**Keywords:** Phospholipid, Nanomechanics, Langmuir-Blogget film, Atomic force microscope, Indentation force curve

## Abstract

**Background:**

Phospholipid, a main component of cell membrane, has been explored as a model system of the cell membrane and temporary scaffold materials in recent studies. The mechanical properties of phospholipid layers are essentially interesting since it is involved in several biological processes.

**Results:**

Here, the nanomechanical properties such as indentation force, adhesion force and DMT (Derjaguin-Müller-Toporov) modulus of 1,2-distearoyl-sn-glycero-3-phosphocholine (DSPC) Langmuir-Blodgett (LB) films were analyzed layer by layer with Atomic Force Microscope (AFM) under deionized water condition.

**Conclusions:**

The penetration distances in the indentation force curves are equal to the thicknesses of phospholipid films, and the yield forces of DSPC LB films in deionized water are smaller than that of similar lipid films in buffered solutions due to the influence of ions. Moreover, the DMT modulus of upper layer DSPC LB film is different from that of monolayer DSPC LB film due to the influence of their different substrates. Our results suggest that environment such as surrounding ions and substrate will strongly influence the measured nano-mechanical properties of the lipid bilayer, especially that of the down layer.

**Graphical Abstract:**
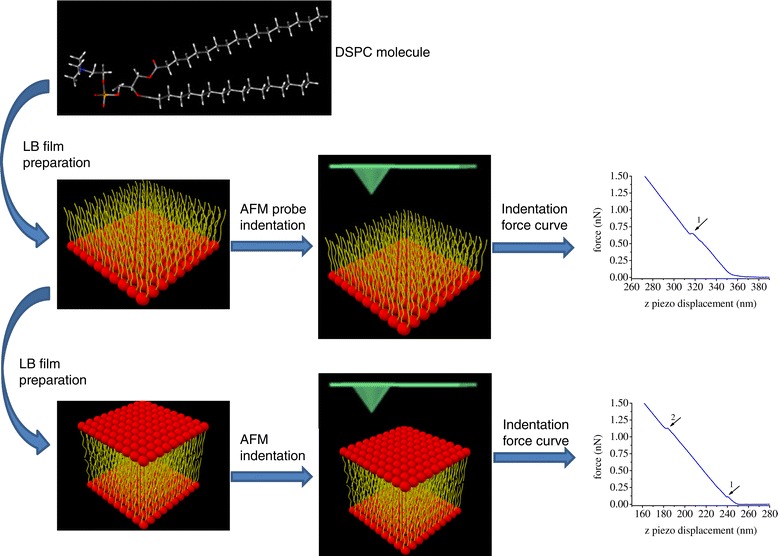
A process about the exploration of nanomechanics of DSPC LB film.

## Introduction

Membranes are essential for life as they are the only means by which biochemical reactions of closed compartments of a living cell can be shielded from the environment [1]. Monolayer and bilayers of lipid not only constitute the matrix of the cell membrane [2], but also could be used as models of the cell membrane [1,3] which makes it convenient to study these models *in vitro*. Phospholipid has been used as temporary scaffold materials to provide mechanical support for the vessel wall [4], and their two-dimensional assemblies of bilayers have been explored as biomimetic analogues of cell membranes [5].

The biological relevant function of lipid layer has aroused interest in exploring its mechanical properties because these mechanical properties govern several biological processes [6], e.g., osmotic shrinkage and swelling of cells [7]. Interacting with the first lipid layer and further permeating through the second layer of cell bilayer membrane, substances can execute biological functions such as exchanging of nutrients, ions [8], and signalling [9] between cells and external environment. Therefore, the mechanics of both the first lipid layer and the second layer are closely related to the biological functions of cells. Nanomechanical properties have been explored by Atomic Force Microscope (AFM), micropipet or other techniques in phospholipid microbubbles [10], vesicles [7] and living cells [11]. Recently, studies on nanomechanical properties of phospholipid layers have been focused by experimental and numerical analysis [12]. The environmental effects on the nanomechanics of lipid layers caused by ions [13], temperature [14], proteins [15], molecular chain unsaturation and headgroup type [16] have been explored in detail with the method of AFM or single molecule force spectroscopy (SMFS) [17]. Moreover, the recent developments in AFM monitoring bilayer alteration and remodeling have been reviewed by Sandrine M. and his coworkers [18] due to the significance of the lipid layers in modeling of biomimetic systems.

Although there are some literatures about the nanomechanical properties of several phospholipids in salt solution reported, the indentation force curve of monolayer, upper layer or lower layer of bilayer phospholipid prepared on substrate has not been reported yet. The reason might be that the nanomechanics of lipid layers are difficult to be obtained [12], especially in low ionic strength concentrations [13,15]. Moreover, the properties of the upper layer and lower layer of the bilayer would be different under the influence of ions or substrate, but they have not been analyzed to our knowledge.

In order to address this issue, DSPC was selected in our experiment. DSPC has been explored extensively since it is one of the main components of cell membranes and in solid state at room temperature. Fresh monolayer and bilayer LB films of DSPC were prepared, and their nanomechanical properties were measured layer by layer using AFM probes with smaller spring constant parameter (0.02 N/m) in deionized water. The indentation force curves of DSPC monolayer, upper layer and lower layer LB films were obtained for the first time. The analysis shows that the obtained penetration distances of the indentation force curves are equal to the thickness values of DSPC LB films, while the DMT modulus [19] of DSPC upper layer is different with that of monolayer films.

## Experimental sections

**Figure 1:**
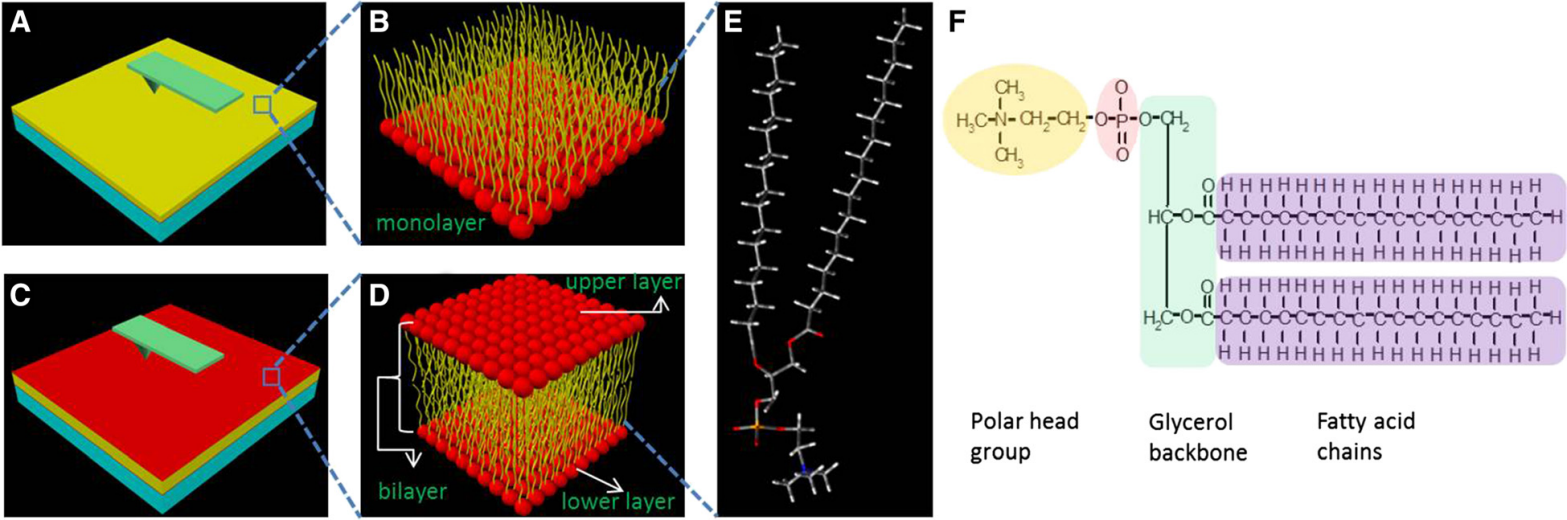
**Schematic cartoon of the AFM tip interacting with DSPC LB film, where the hydrophobic side and hydrophilic side of the film are illustrated in yellow and red respectively. (A)** Interaction between AFM tip and DSPC monolayer LB film. **(B)** Monolayer of DSPC LB film, where the red ball and yellow bent rods represent the hydrophilic head and the hydrophobic tails of the DSPC molecule respectively. **(C)** The interaction of the AFM tip and the bilayer LB film of DSPC. **(D)** Bilayer LB film of DSPC. **(E)** Molecular structure of DSPC. **(F)** Structural formula of DSPC, where the choline and phosphate group constitute the hydrophilic head group. The twin saturated alkyl tails, connected to the polar head group through the glycerol backbone, constitute the hydrophobic tails.

The twin saturated alkyl tails of DSPC molecule and the polar phosphatidylcholine headgroup are connected each other through a glycerol linker [20], which constitute the long hydrophobic alkyl tails and the hydrophilic headgroup. Langmuir–Blodgett (LB) [21] technique was used to prepare the phospholipid films, and AFM was used to characterize these obtained LB films. The interactions between AFM probe tip and DSPC LB films are described in Figure 1A and 1C, where the hydrophobic side and hydrophilic side of the film are illustrated in yellow and red respectively. The detail structures of these monolayer and bilayer LB films are depicted in Figure 1B and 1D, and the molecular structure of DSPC is revealed in Figure 1E and Figure 1F where the choline, phosphate group, glycerol backbone and the saturated alkyl tails with 18 repeat carbon units (-CH_2_) [20] are clearly described.

### Materials

1,2-distearoyl-sn-glycero-3-phosphocholine (DSPC) and 1,2-dilauroyl-sn-glycero-3- -phosphocholine (DLPC) were purchased from Avanti polar lipids Inc. (Alabaster, AL). Chloroform was purchased from Tianjin Kermel Chemical Company Ltd. Fresh ultrapure deionized water with resistivity of 18.23 MΩ · cm was produced using GT-8L ion exchange system. All substrates used in the experiment were mica with freshly cleaved surface (Ted Pella, Inc.).

### LB film preparation

Unique deposition technique such as LB deposition is generally used to obtain films with controlled packing density of molecules over a wide range, which is not easily accomplished with chemisorbed or other assembling methods [20]. In our experiment, the assembled monolayers and bilayers of phospholipid films were prepared in a computer controlled LB trough (μ trough, Kibron Co., Helsinki, Finland) [22] which monitors surface pressure using a Whilhelmy wire. 25 μL 0.5 M phospholipid chloroform solutions were applied onto the surface of deionized water subphase (about 70 mL) with a microsyringe. After 20 minutes, the phospholipid film was compressed with two barriers at 3 cm^2^/min compression rate until surface pressure reached 45 mN/m (compact LB films could be obtained at this surface pressure though it is smaller than the collapse surface pressure of the used phospholipid), and then the two barriers were released to the sides of the trough at the speed of 5 cm^2^/min. After 3 times of compression, monolayer phospholipid film was deposited onto a newly cleaved mica substrate (1cm × 1 cm, previously immerged into water subphase) with a lifting rate of 1.5 mm/min. The second layer of phospholipid was transferred onto the monolayer film with a dipping rate of 1.5 mm/min when the monolayer film was dried 10 minutes in air. The compensation rate of 3 cm^2^/min was used to keep a constant pressure of the interface during LB film deposition process by the aid of a Whilhelmy wire to monitor the sample pressure. This compensation speed of 3 cm^2^/min is merely a maximum speed, which could be auto-tuned to adapt to the sample raising speed in order to keep the sample pressure constant. The configuration of the prepared phospholipid bilayer films is similar to that of cell membrane in nature [8]. In the end, the phospholipid bilayer was brought out of the subphase with the speed of 10 mm/min, and AFM measurements were carried out right after the film preparations.

### AFM characterization

AFM characterizations were carried out in contact mode under deionized water using an Agilent AFM/STM 5500 microscope with a Multi-purpose small scanner with low coherence laser (N9520A, Agilent Technologies, Inc. USA). The cantilever was a 100 μm long and 106 μm wide silicon nitride triangular (OMCL-TR400PSA from Olympus, Japan), with a nominal spring constant of 0.02 N/m and tip radius less than 20 nm. The imaging frequency was fixed to 1.0 Hz, and the resolution of images was 512 × 512 pixels per image. Monolayer and bilayer films were confirmed with the Agilent AFM/STM 5500 microscopy at first, and then the indentation force curves were recorded at different sites of the phospholipid LB films without defect. The sweep duration of AFM tip approaching and retracting, and pre-sweep delay time were all set as 1.0 second. The adhesion and DMT modulus of the samples were studied with another AFM of MultiMode® 8 from Bruker AXS. All recorded images and indentation force curves were analysed using the commercial Scanning Probe Image Processor (*SPIP*™) software (image metrology ApS, version 5.13, Lyngby, Denmark). All indentation force curves were processed with force curve analysis module, and Hertz sphere-on-flat model was chosen to fit the force curves. The Poisson’s ratio was assumed to be 0.5 (highly elastic), which is in agreement with related studies.

## Results and discussion

### Monolayer and bilayer of phospholipid films

Monolayer and bilayer phospholipid films were prepared with LB technique since it is convenient to prepare films with controllable layers, which is different from the experiments where films were prepared by dropping phospholipid solutions onto substrates [13,14] or absorbed on AFM probe tips [15]. The monolayer and bilayer phospholipid films were then used to analyze their mechanical properties layer by layer in this paper.

#### Monolayer

**Figure 2:**
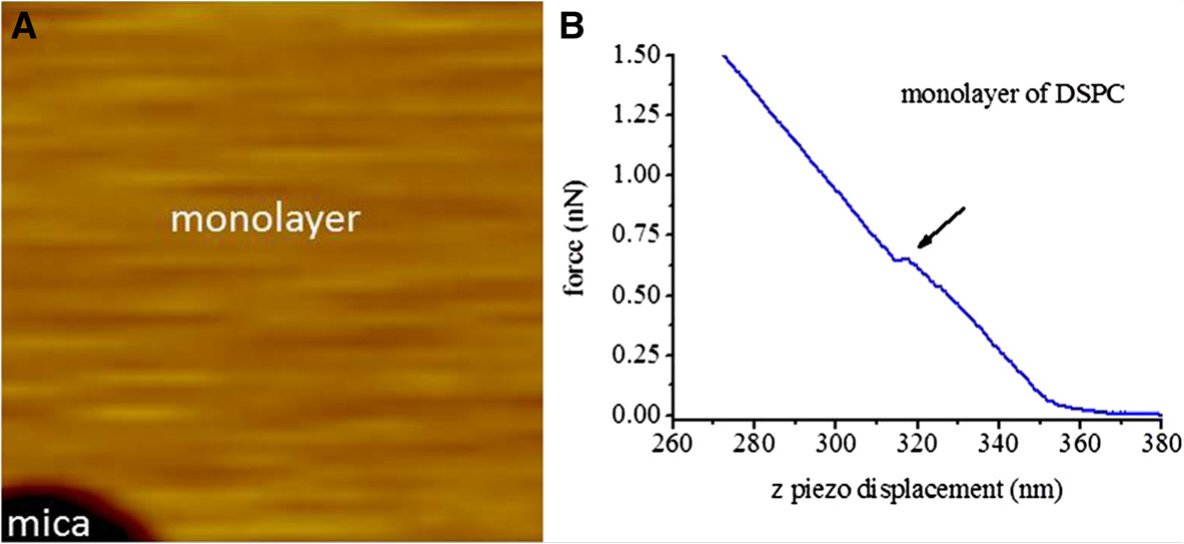
**AFM results of DSPC monolayer LB film. (A)** Topography of DSPC monolayer LB film with 400 nm × 400 nm area. **(B)** An example of indentation force curve of DSPC monolayer LB film in deionized water.

Figure 2 shows AFM measurement results of freshly made DSPC monolayer LB film on mica substrate (marked in Figure 2A). According to the topography image shown in Figure 2A, it is obvious that there is a defect on the prepared DSPC monolayer LB film. This defect can be used to distinguish assembled molecular film from the substrate in future work. Indentation force curves of DSPC monolayer film were obtained at different sites of the film without defect, and an example of indentation force curve is described in Figure 2B where there is a yield threshold [13] denoted by a black arrow. The break through force [13] at the yield point (f_m_) is 0.85 ± 0.52 nN and penetration distance (w_m_: width of the jump) is 2.24 ± 1.01 nm counted from 510 curves. This statistical penetration distance of the DSPC monolayer film calculated from indentation force curves represents the thickness of the film, which is in agreement with that of DSPE (DSPE has the same twin alkyl tails with that of DSPC) monolayer in water (about 2 nm) measured by modified AFM probe [23]. In addition, the break through force at the yield threshold and the measured thickness values with AFM are related to several experimental parameters, such as the cantilever spring constant, the shape and size of the used AFM tip. These mentioned parameters are diverse from each other actually, which results in large error ranges. Compared with the data ranging between 4nN to 10 nN [13], the error ranges of our break force and penetration distance are acceptable based on the measurements of 200 or 510 force curves.

In order to validate the indentation force curves of DSPC monolayer LB film, monolayer film of another phospholipid (DLPC) was researched with the same method as that of DSPC monolayer film in both sample preparation and sample characterization. DLPC has the same basic molecular structure as that of DSPC. DLPC has 12 repeat carbon units (-CH_2_) while DSPC has 18 repeat carbon units [20], and then the length of DLPC molecule is shorter than that of DSPC molecule. Then the layers of the two phospholipids were expected to be of distinct mechanical properties at nano (or pico) Newton level.

**Figure 3:**
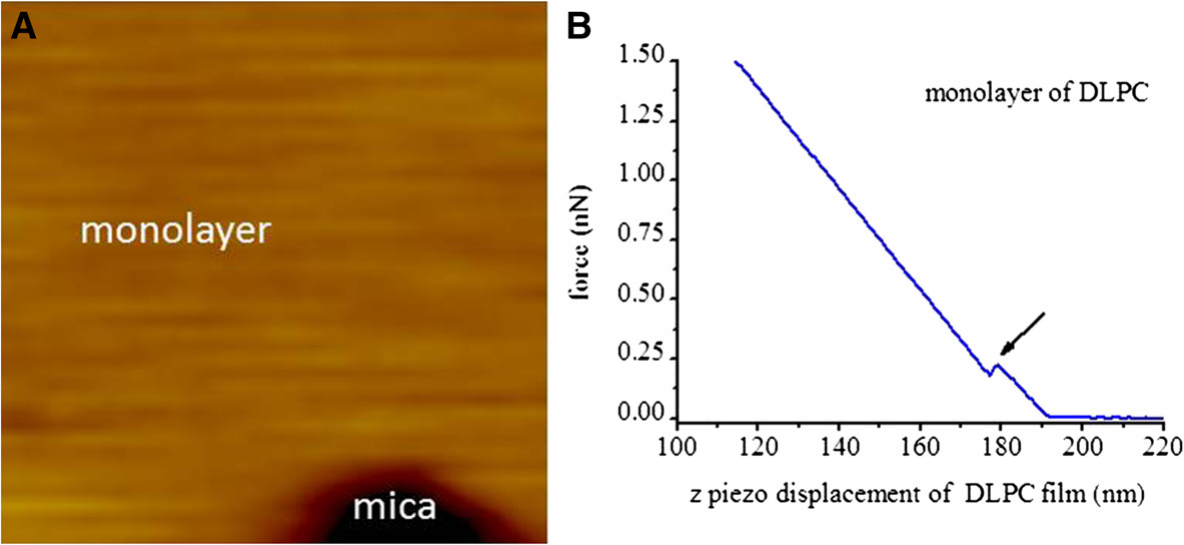
**AFM results of DLPC monolayer LB film. (A)** Topography of DLPC monolayer LB film with 400 nm × 400 nm area, **(B)** An example of indentation force curve of DLPC monolayer LB film in deionized water.

AFM experiment results of DLPC are expressed in Figure 3A and Figure 3B. The defect in the topography image (Figure 3A) of DLPC monolayer film was used to distinguish the film from the substrate topography (marked in Figure 3A), and the indentation force curves were collected at different sites of the film. The indentation force curve of the film in AFM tip approaching process is similar to that of DSPC (shown in Figure 2B), where there is also a yield threshold marked by a black arrow. The break through force at the yield point is 0.41 ± 0.26 nN and penetration distance is 2.33 ± 0.87 nm counted from 510 curves. The statistical yield force of DLPC monolayer is smaller than that of DSPC monolayer, which states that DLPC monolayer, composed of molecules with shorter alkyl tails (12 repeat carbon units), is weaker than that of DSPC (18 repeat carbon units) monolayer. In other words, DSPC monolayer is more difficult to be penetrated.

It will be found that the penetration distances of phospholipid films are smaller than that of films’ theoretical thickness, which is mainly due to the deformation of the phospholipid molecules when they are interacting with AFM tip during the penetrating process in our experiment. Moreover, the deformation of DSPC film (in ordered-phase) would be different from that of DLPC film (in fluid-phase) under experimental temperature of 28°C when their phase transition temperature are considered (phase transition temperature of DSPC is 57.5 ± 0.5°C while that of DLPC is -1°C [24,25]), which could be helpful in understanding the experiment result that no obvious penetration distance difference were obtained between DSPC and DLPC.

#### Bilayer

**Figure 4:**
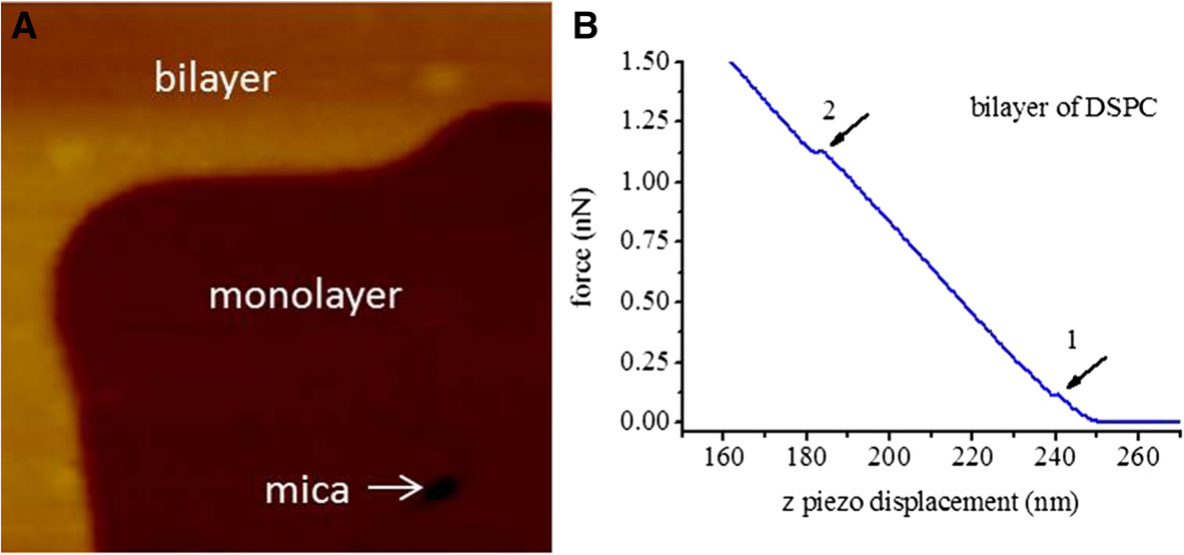
**AFM results of bilayer DSPC LB film. (A)** Topography of bilayer DSPC LB film with the area of 400 nm × 400 nm. **(B)** An example of indentation force curve of DSPC bilayer LB film in deionized water.

Fresh LB films of DSPC bilayer were investigated in this experiment, even if it has been reported that no changes of bilayer (distearoylphosphatidylethanolamine (DSPE), lactosylethanolamine (MGDG) and dioleoylethanolamine (DOPE)) in the AFM images or force data were observed up to 14 hour after deposition with LB trough onto mica substrate at the surface pressure of 25 mN/m in contact mode under triply distilled water [23]. The topography image for DSPC bilayer LB film is shown in Figure 4A, in which monolayer and bilayer regions are marked, and AFM force curves were collected in the region of DSPC bilayer. Indentation force curves of bilayer DSPC LB film in approaching process (shown in Figure 4B) are different from that of DSPC monolayer (shown in Figure 2B), where there are two yield thresholds marked by arrow 1 and arrow 2. The two yield thresholds mean that the upper and lower layers were penetrated in turn when AFM probe tip approached to the surface of DSPC bilayer film, which is different from the previous studies where each yield threshold stands for the penetration of a bilayer [13,15] (their sample are composed of several layers of bilayer phospholipid film). The break through forces at the yield points 1 and 2 are 0.38 ± 0.28 nN (f_u_) and 0.79 ± 0.39 nN (f_d_) respectively, and the corresponding penetration distances are 1.99 ± 0.80 nm (w_u_: width of the first jump) and 2.65 ± 1.17 nm (w_d_: width of the second jump) counted from 200 curves.

The penetration distances measured both at the first yield threshold and the following one, which represent the thicknesses of the upper and lower layers, are close to the width of jump (w_m_) in the case of DSPC monolayer. This result is reasonable since each of the penetration distance demonstrates the thickness of a single layer of DSPC LB film. The value of w_u_ plus w_d_ is about 4.64 nm, the total penetration distance of DSPC bilayer, which is the detected thickness of DSPC bilayer in our experiment. This detected thickness of DSPC bilayer is not only in agreement with the thickness of DSPC bilayer measured by modified AFM tip (4.4 nm) [26], but also consistent with the result measured by X-ray (4.7 nm) [27]. Moreover, the D-value (f_d_-f_u_) of the break force between the lower and upper layers is about 0.41 nN, which is almost equal to the break through forces of upper layer (f_u_: 0.38 ± 0.28 nN) of DSPC LB films. This result means that the lower layer is as strong as that of the upper layer to be penetrated even if it is not expected since there is hydrophobic surface upside for lower layer DSPC LB film, while there is hydrophilic surface upside for upper layer. The possible reasons for that might be the water [28] and the hydration layer (formed between the mica surface and the hydrophilic surface of lipid films) [19] can affect the penetration forces.

The yield threshold force of DSPC bilayer (lower layer covered with upper layer) obtained in this work (f_d_ =0.79 ± 0.39 nN, this force was used to represent the force needed to penetrate bilayer DSPC LB film since it was the force used to penetrate the lower layer of bilayer DSPC LB film after the AFM tip penetrated the upper layer of bilayer DSPC LB film.) is smaller than that of similar phospholipid film (1,2-dimyristoyl-sn-glycero-3-phosphocholine (DMPC) or 1,2-dipalmitoyl-sn-glycero-3-phosphocholine (DPPC), Figure Eight A in reference [14]) at the same temperature of 28°C, which could be explained by the effect of ions. The effects of ions, molecular structure and temperature on the nanomechanics of DLPC, DMPC and DPPC bilayers have been studied by Sergi and his coworkers [13,14], where all solutions used were set at pH =7.4 with 10 mM Hepes/NaOH. The basic molecular structures of DLPC, DMPC and DPPC [13] are the same as that of DSPC, which only differ in the number of –CH_2_ groups in the fatty acid chains. The number of –CH_2_ groups are 12, 14, 16 and 18 for DLPC, DMPC, DPPC and DSPC respectively. The yield threshold forces of DMPC are about 2 nN (Figure Eight A in reference [14]) in buffered water solution (f_l_) at 30°C, which is much smaller than that in buffered high ionic strength solution (about 4.7nN (f_h_), f_l_ is equal to 0.43 times of f_h_). The same phenomenon was also found in the case of DPPC and DLPC bilayers at different temperatures by Sergi et. al., which explores the significant impact of ions on the yield threshold force of lipid film. This conclusion could be used to explain the small yield threshold force of DSPC bilayer (lower layer covered with upper layer) obtained in our work (0.79 ± 0.39 nN) compared with that of DMPC or DPPC at the same temperature (Figure Eight A in reference [14]) considering that our experiment was conducted under the deionized water condition.

Phenomenon should be pointed out that the slopes of the indention force curves changed very little after AFM tip having penetrated phospholipid films both in our experiment (Figures 2, 3 and 4) and in references [13,14]. The major reason for that could be that AFM tips with different spring constant cantilevers fit for samples with different elastic modulus ranges, and softer cantilevers suit for lower elastic modulus samples [29]. It has been reported that the sensitivities of AFM probe cantilevers can be affected by sample hardness [30], and then, the cantilever with 0.02 N/m spring constant were selected for phospholipid films imaging and not suitable for measurement of stiffer mica in our measurement.

### Mechanical property of phospholipid film at different layers

The mechanical property of the thin film will be influenced by its substrate, and the influence of the substrates on nanomechanical property of thin film has been studied in detail [31]. For example, the Young’s modulus of thin film on different substrates increased with the hardness of substrate [32]. Moreover, fitting model of force curve was suggested to be modified to reduce the influence of substrate [33]. Then, the influence of substrate should be minimized to obtain the nature of phospholipid film in this study. The substrate of phospholipid lower layer of LB film is mica, while the substrate of the upper layer is the lower layer composed of the same kind of molecules for bilayer LB films. The nature of phospholipid film will be closer to that of upper layer, therefore a bilayer was needed as a model to be studied. With a MultiMode® 8 AFM instrument from Bruker AXS with a NanoScope V controller and NanoScope (version 8.10) software, the adhesion and DMT modulus of bilayer were explored. These elastic moduli mappings based on novel AFM instrument enable us to study surface stiffness with nanoscale resolution in a quantitative way [34]. The AFM tip is approximately considered as spherical at the very end, and then DMT (Derjaguin-Müller-Toporov) model [35] is thought as a suitable model [34] to analysis AFM tip and lipid film at the moderate adhesion levels. The DMT modulus is used to measure the Young’s modulus for materials [19]. It has been reported that the DMT modulus values obtained with MultiMode® 8 AFM at different working modes are in very good agreement with each other (HarmoniX DMT modulus mapping mode and Peak Force Tapping DMT modulus mapping mode) [34]. In order to calculate the modulus of the lipid film, the measured force indentation curves were processed with the help of DMT model where the interactions of adhesion were taken into account [36]. The force (F_l_) indentation curves were fitted with Young’s modulus (E), radius of indenter (R_indenter_) and sample surface (R_surface_), indentation depth (i), Poisson’s ratio (υ) and adhesion force (F_pull-off_) using following equation:

$$ {F}_l=\frac{4}{3}{E}^{*}\sqrt{R^{*}}{i}^{\raisebox{1ex}{$3$}\!\left/ \!\raisebox{-1ex}{$2$}\right.}+{F}_{pull- off}, $$

Where

$$ \raisebox{1ex}{$1$}\!\left/ \!\raisebox{-1ex}{${R}^{*}$}\right.=\raisebox{1ex}{$1$}\!\left/ \!\raisebox{-1ex}{${R}_{indenter}$}\right.+\raisebox{1ex}{$1$}\!\left/ \!\raisebox{-1ex}{${R}_{surface}$}\right.\kern0.5em \mathrm{and}\kern1.5em {E}^{*}=\raisebox{1ex}{$E$}\!\left/ \!\raisebox{-1ex}{$\left(1-{\upsilon}^2\right)$}\right. $$

**Figure 5:**
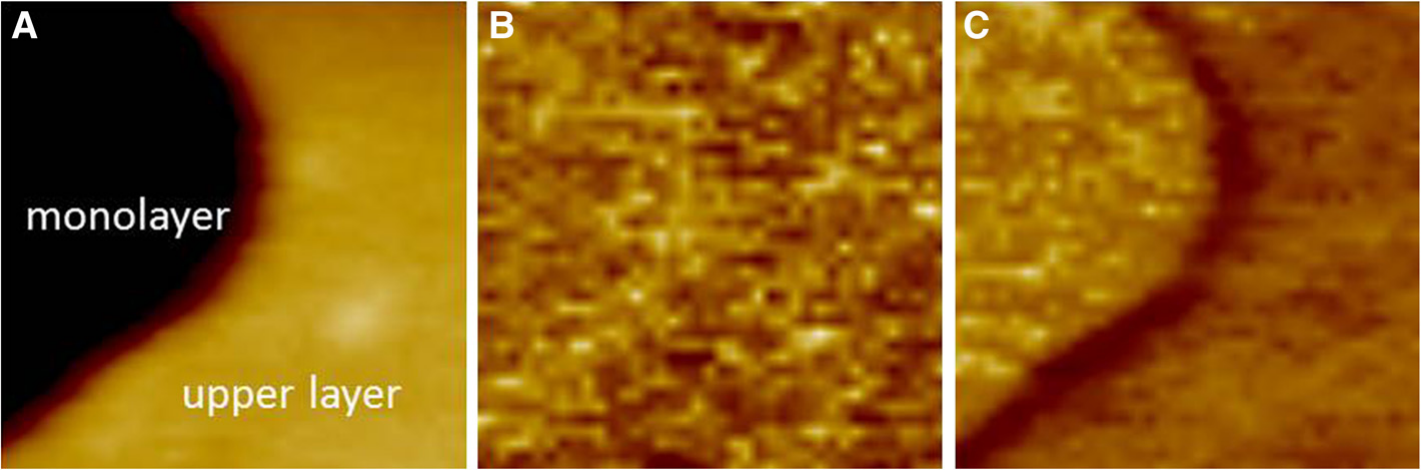
**AFM images of bilayer DSPC on mica substrate obtained with MultiMode® 8 with the area of 150 nm × 150 nm. (A)** Topography image of bilayer DSPC LB film on mica. **(B)** Adhesion of the bilayer film. **(C)** DMT modulus of the bilayer film.

Figure 5A, B, and C respectively described the topography, adhesion and DMT modulus of the monolayer DSPC LB film and the upper layer of DSPC bilayer film. Figure 5A clearly shows the upper layer and the monolayer (marked in Figure 5A) of DSPC bilayer film. The adhesion of monolayer is almost equal to that of the upper layer in Figure 5B, which means that their properties are consistent with each other. This result is expected since the monolayer and the upper layer are assembled with the same material. Considering that monolayer DSPC LB film has hydrophobic functional groups upside while that of upper layer DSPC LB film has hydrophilic groups upside, people with different points of view may expect different adhesion results. It might be that the layer of hydration, formed between the hydrophilic molecules’ terminations and the substrate, reduced the adhesion of the lipid film to the substrate [19]. More importantly, it has been reported recently that the adhesion properties of the first few layers is dominated by the surface potential of substrate [37]. Considering the above discussion, the difference of adhesion could be smeared out.

According to Figure 5C, DMT modulus value of monolayer is calculated to be 68 ± 10 MPa (E_m_), and that of upper layer is 48 ± 12 MPa (E_u_), which are in the same order of magnitude as the Young’s modulus of DOPC/DPPC (1:1, mol/mol) membranes (19.3 MPa and 28.1 MPa for the liquid and gel phases respectively) [38]. The higher DMT modulus of DSPC LB films in our experiment, compared with the Young’s modulus of DOPC/DPPC films in liquid or gel phases, are reasonable since DSPC LB films are in solid phase under experimental temperature of 28°C (phase transition temperature of DSPC is 57.5 ± 0.5°C [24]).

The difference of DMT modulus between DSPC upper layer and monolayer is about 20 MPa, which could be resulted from their different substrates, since they are made of the same kind of molecules. This result indicates that the Young’s modulus of monolayer phospholipid film on soft lower layer or mica (with young’s modulus of 65.7 GPa [39]) increased with the hardness of substrate, which agrees with literature [32]. Moreover, the adhesion property of the upper layer is almost equal to that of the monolayer analyzed from Figure 5B. These observations imply that the property of the upper layer is similar to that of the monolayer, but their property is not completely the same under the influence of their different substrates. The different property of bilayer and monolayer in our experiment indicates that the nature of DSPC LB film is closer to that of the upper layer, and this is why we focus on them. Especially, ions or other reagent in solvent used in experiment will have an influence on the property of the lipid layers, and the influence on the bilayer might be different from that on the monolayer since the cations or small molecules need more energy to pass through the bilayer than that of the monolayer.

## Conclusions

In our experiment, the assembled monolayer and bilayer films composed by DSPC phospholipid molecules were prepared with the assistance of LB technique under the controllable conditions. Then, their nanomechanics properties were characterized layer by layer with AFM in contact mode under deionized water condition for the first time. The nanomechanics of DSPC monolayer, upper layer and lower layer of bilayer were analyzed and compared. It is found that though the adhesion of upper layer phospholipid is consistent with that of monolayer made from the same material, the DMT modulus of upper layer is closer to the nature of DSPC LB film. All obtained data of the penetration distances for DSPC represent the thicknesses of the lipid films as expected, and the mean yield force of DSPC LB bilayer in deionized water obtained in this work is smaller than that of similar lipid bilayers such as DMPC and DPPC in buffered water in the previous work due to the effect of ions. The success of exploring the nanomechanics of the phospholipid in deionized water is helpful to analyze the influence factors (cations or other chemicals) on the phospholipid membranes.
